# Virally and physically transgenized equine adipose-derived stromal cells as a cargo for paracrine secreted factors

**DOI:** 10.1186/1471-2121-11-73

**Published:** 2010-09-23

**Authors:** Gaetano Donofrio, Antonio Capocefalo, Valentina Franceschi, Giorgio Morini, Maurizio Del Bue, Virna Conti, Sandro Cavirani, Stefano Grolli

**Affiliations:** 1 Dipartimento di Salute Animale, Sezione di Malattie Infettive, Facoltà di Medicina Veterinaria, via del Taglio 10. 43100 Parma, Italy; 2 Dipartimento di Salute Animale, Sezione di Clinica Ostetrica e Riproduzione Animale, Facoltà di Medicina Veterinaria, via del Taglio 10. 43100 Parma, Italy; 3 Dipartimento di Salute Animale, Sezione di Clinica Chirurgica Veterinaria e Medicina d' Urgenza, Facoltà di Medicina Veterinaria, via del Taglio 10. 43100 Parma, Italy; 4 Dipartimento di Produzioni Animali, Biotecnologie Veterinarie, Qualità e Sicurezza degli Alimenti, Sezione di Biochimica Veterinaria, Facoltà di Medicina Veterinaria, via del Taglio 10. 43100 Parma, Italy

## Abstract

**Background:**

Adipose-Derived Stromal Cells have been shown to have multiple lineage differentiation properties and to be suitable for tissues regeneration in many degenerative processes. Their use has been proposed for the therapy of joint diseases and tendon injuries in the horse. In the present report the genetic manipulation of Equine Adipose-Derived Stromal Cells has been investigated.

**Results:**

Equine Adipose-Derived Stromal Cells were successfully virally transduced as well as transiently and stably transfected with appropriate parameters, without detrimental effect on their differentiation properties. Moreover, green fluorescent protein alone, fused to *neo *gene, or co-expressed as bi-cistronic reporter constructs, driven by viral and house-keeping gene promoters, were tested. The better expressed cassette was employed to stably transfect Adipose-Derived Stromal Cells for cell therapy purposes. Stably transfected Equine Adipose-Derived Stromal Cells with a heterologous secreted viral antigen were able to immunize horses upon injection into the lateral wall of the neck.

**Conclusion:**

This study provides the methods to successfully transgenize Adipose-Derived Stromal Cells both by lentiviral vector and by transfection using optimized constructs with suitable promoters and reporter genes. In conclusion these findings provide a working platform for the delivery of potentially therapeutic proteins to the site of cells injection via transgenized Equine Adipose-Derived Stromal Cells.

## Background

Defects of the cartilage, as well as of tendons, have been known to be poor healing injuries, which treated by conventional surgical or pharmacological methods often do not lead to complete structural and functional tissue recovery and regeneration. For this purpose, transplantation of autologous or allogeneic pluripotent or terminally-differentiated cells that are expanded *ex vivo *have been attempted to cure such defects [1]. The majority of research concerning mesenchymal stem cells (MSC) has been done on rodents or humans. Small experimental animals can be used to research the principles of stem cell transplantation therapy, but prior to transferring this technology to the therapy it is important to introduce it to a large-animal model, which is bio-mechanically more relevant to humans. Athlete equines are considered valuable large-animal experimental models for osteoarticular and tendon disorders, since most of them can be considered similar to the corresponding human pathologies with regard to pathogenetic mechanisms and clinical outcomes. As a consequence, the application of cell therapy in the horse has attracted considerable interest not only as a new therapeutic approach for equine musculoskeletal diseases but also because it can provide numerous inputs for making progress in clinical application of MSC to human medicine, especially in musculoskeletal health problems [2]. Joint disease, and specifically osteoarthritis (OA), is one of the most prevalent and debilitating diseases affecting both humans and horses. The equine as animal model is a potential donor of relatively large amounts of tissue and cells, and consequently leads to sufficient yield of isolated MSCs, providing further opportunity to use them in numerous *in vitro *and *in vivo *experiments.

Some of the challenges in stem cell research are the expansion, propagation, and genetic manipulation of functional adult stem cells. The clinical application of neural stem cells will be limited by logistic and ethical problems associated with their isolation and by potential immunologic incompatibility due to the requirement for allogeneic transplantation. On the contrary, adult stem cells derived from mesodermal sources, such as bone marrow and adipose tissue, can be obtained from patients with greater ease and because autologous transplantation obviates immunologic incompatibilities.

Although most of the pioneering studies have been performed on bone marrow-derived MSC, recently adipose tissue has attracted interest as a source of multipotent cells for therapeutic applications. Adipose tissue is ubiquitous and uniquely expandable. Most patients possess excess fat that can be harvested making adipose tissue an ideal source for clinical research. Adipose-Derived stromal cells (ADSCs) have been examined as an alternative to bone marrow stromal cells and have been shown to be comparable [3]. Here, the transient and stable genetic manipulation of Equine ADSCs (EADSCs) using viral and non viral systems was evaluated, thus providing the proof of principle of the ability of EADSCs to potentially deliver bio-active factors in a paracrine manner to the site of the cell injection; moreover, the secreted antigen could metastatically, i.e. by blood stream, be delivered to other body sites following cell homing.

## Results

### hCMV promoter is strongly activated in EADSCs

**Figure 1:**
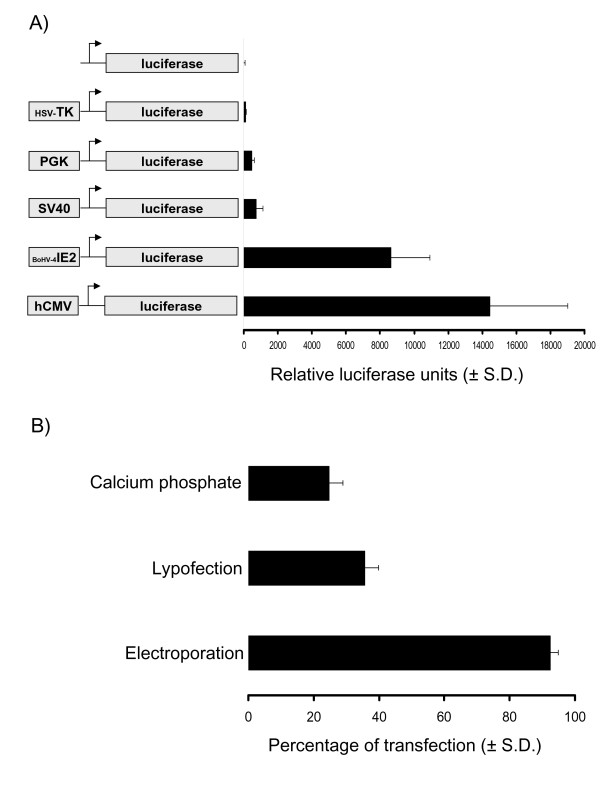
**Promoters activation in EADSCs**. A) Transiently transfected EADSCs with constructs containing promoter in front of the luciferase reporter gene or pGL3 empty vector (diagrams not on scale) as a control, at 24 h post transfection. The results are expressed as the mean relative Luciferase units after normalizing with efficiency of transfection. Each reaction was done in quadruplicate, and each point represents the average ± standard deviation from three experiments. B) Efficiency of EADSCs transfection obtained with different transfection methods, using pEGFP-C1 as a reporter plasmid. Each transfection method was done in quadruplicate, and each point represents the average ± standard deviation from three experiments. EGFP was monitored by fluorescence microscope and quantified by flow cytometry.

Although epigenetic regulation of a transgene is very important, transgene transcription is strongly dependent on the type of promoter employed to drive the expression of a desired monocistronic or polycistronic open reading frame (ORF). Therefore, several promoters were tested in a transient transfection assay. To be able to quantify promoters transactivation by the pool of transcription factors made by EADSCs, Herpes Simplex Virus Timidine Kinase (HSV-TK), Phosphoglycerokinase (PGK), Simian Virus 40 (SV40), Bovine Herpesvirus 4 Immediate Early (BoHV-4 IE2) and human Cytomegalovirus Immediate Early (hCMV) promoters were sub-cloned in front of the pGL3 vector luciferase reporter gene. The generated constructs (Fig. 1), as well as pGL3 empty vector, used as a control, were co-transfected with pTK-Renilla for data normalization into EADSCs. Twenty-four hours post-transfection, cells were lysed and luciferase activity was measured. hCMV gave the best expression (Fig. 1A), thus it was used for further experiments. Although this experiment gave good information about the type of promoter better transactivated by EADSCs transcriptional machinery, no information could be achieved in terms of efficiency of transfection, which is tightly dependent on the method employed to transfect cells. Efficiency of transfection of EADSCs was measured comparing 3 different methods of transfection. EADSCs were transfected by calcium phosphate precipitation, lipofection and electroporation with pEGFP-C1 plasmid containing the hCMV promoter, which was shown to be nicely activated into EADSCs, and the enhanced green fluorescent protein (EGFP) as a reporter gene. Transfected cells were analyzed at 48 h post transfection by fluorescence microscopy and flow cytometry for EGFP-expressing cells and the ratio between transfected and untrasfected cells was calculated on the base of three repeated experiments. Electroporation gave the best efficiency of transfection, reaching the ~95%, respect to calcium phosphate precipitation and lipofection (Fig. 1B).

### Viral transduction of EADSCs keeps stable transgene expression following differentiation

**Figure 2:**
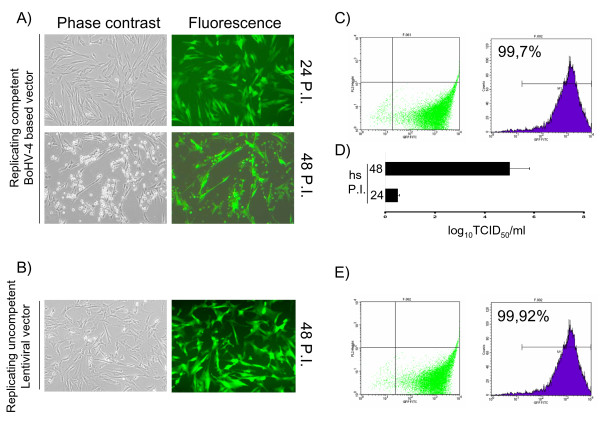
**Viral transduction of EADSCs**. Phase contrast and fluorescence representative images (20×) of BoHV-4-based vector transduced EADSCs at 24 and 48 h post infection (P.I.) (A), along with the transduction efficiency measured by flow cytometry (C) and the viral titer measured at 24 and 48 h post infection (hs P.I.) (D). In B, representative images of lentiviral vector transduced EADSCs at 24 h post infection and respective transduction efficiency measured by flow cytometry (C).

**Figure 3:**
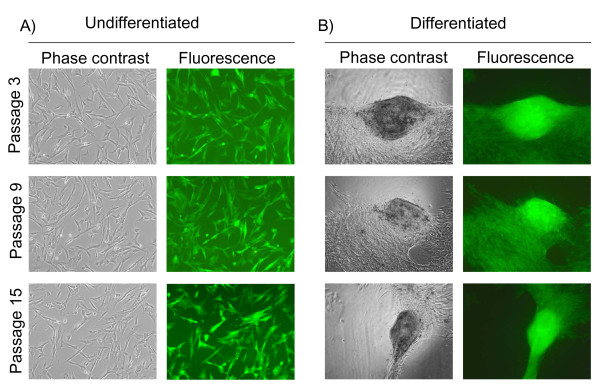
**Viral transduction of EADSCs maintains transgene expression**. Representative phase contrast and fluorescence images (20×) of undifferentiated (A) and differentiated (B) EADSCs at 3, 9 and 15 passages post lentiviral vector transduction.

**Figure 4:**
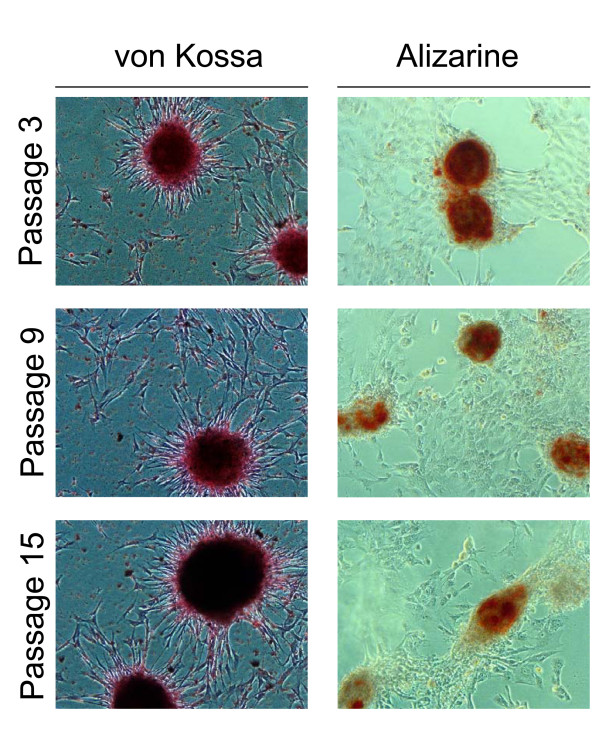
**Viral transduction of EADSCs maintains differentiation properties**. Representative images (20×) of von Kossa and alizarin stained differentiated EADSCs at different passages (3, 9 and 15) post lentiviral transduction.

EADSCs labelling is a very important issue when cell fate in terms of differentiation, bio-distribution and engraftment need to be monitored *in vivo*. As a first attempt to transgenize EADSCs, two different viral vectors were employed: a self-inactivating replicating incompetent third generation lentiviral vector [4,5] expressing EGFP under the control of hCMV promoter and a newly developed bovine herpesvirus 4 (BoHV-4)-based vector [6] expressing EGFP under the control of hCMV promoter. EADSCs were infected with both vectors and efficiency of transduction was monitored at 24 and 48 h post infection. Although both viral vectors transduced EADSCs with a very high efficiency, 99.7% for the BoHV-4-based vector (Fig. 2A and 2C) and 99.92% for the lentiviral vector (Fig. 2B and 2E) respectively, EADSCs were highly permissive for BoHV-4-based vector replication. In fact, a strong cytopathic effect (CPE) developed in EADSCs as far as 48 h post BoHV-4-based vector infection, leading to an high cell free virus titer (Fig. 2D). Lentiviral transduced EADSCs were grown for 15 passages, transgene was constantly expressed for all passages tested (Fig. 3A), and differentiating properties were maintained when EADSCs were induced to differentiate with osteogenic medium (Fig. 3B). The state of differentiation was confirmed with both alizarin and von Kossa staining (Fig. 4).

### Stably transfected EADSCs keep transgene expression following drug selection and differentiation

Starting from the assumption that the hCMV promoter was the best promoter to drive the expression of an heterologous gene in EADSCs and that electroporation was the best physical method to transfect EADSCs, a suitable combination between a reporter gene and a selectable marker integrated into the same vector to stably label and select EADSCs was investigated.

**Figure 5:**
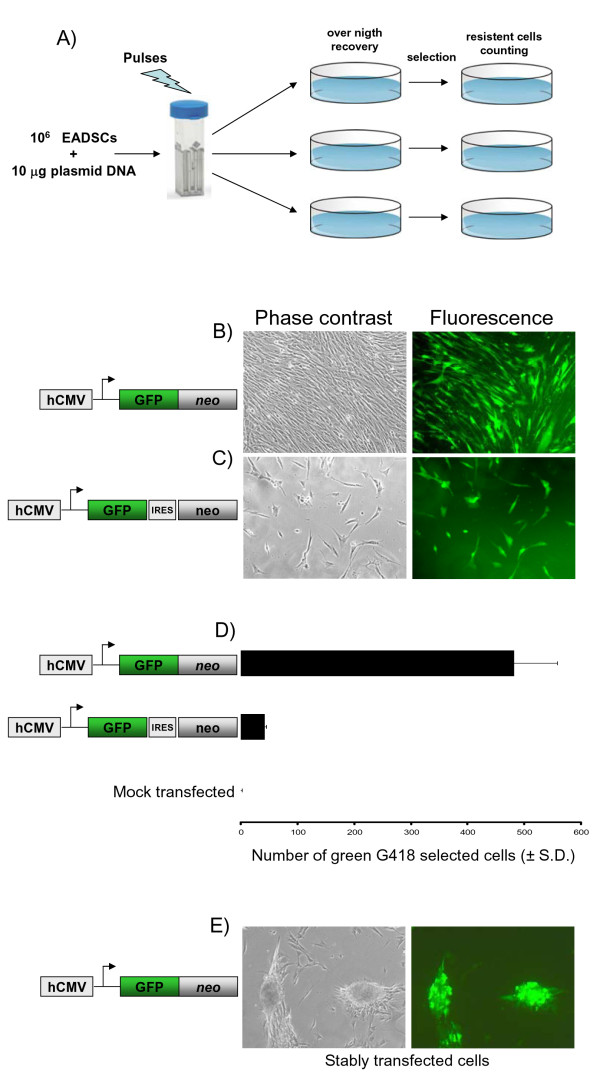
**Generation of stably transfected EADSCs**. A) Flow chart of the method used to obtain a homogeneous population of EGFP stably labelled EADSCs by electroporation and drug selection. Phase contrast and fluorescence representative images (20×) of green G418-selected EADSCs generated with pCMV-EGFP/*neo *fusion peptide (B) and pCMV-EGFP-IRES-*neo *bicistronic vector (C) (diagram not on scale beside the images). D) Generating efficiency of green G418 selected EADSCs obtained with pCMV-EGFP/*neo *and pCMV-EGFP-IRES-*neo *respectively and compared to mock-transfected EADSCs. Each experiment was done in quadruplicate, and each point represents the average ± standard deviation from three experiments. Green G418 EADSCs were monitored by fluorescence microscope and quantified by flow cytometry. E) Phase contrast and fluorescence representative images of differentiated stable green G418 selected EADSCs obtained with pCMV-EGFP/*neo*.

**Figure 6:**
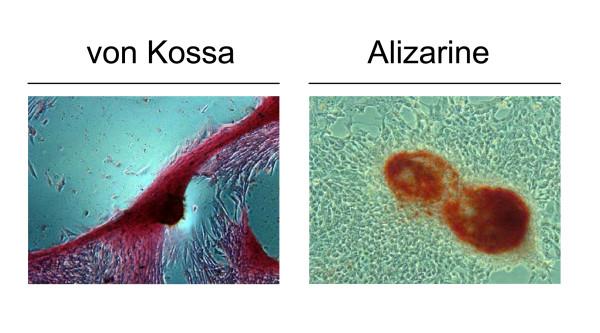
**Stably transfected EADSCs maintains differentiation properties**. Representative images (20×) of von Kossa and alizarin stained stable green G418 selected EADSCs obtained with pCMV-EGFP/*neo*.

As a selectable marker, the *neo *gene (Aminoglycoside 3'-phosphotransferase), due to its low toxicity and large window of concentration, was used. Whereas EGFP, due to its high stability and direct detectability in living cells, was employed as a reporter gene. Although EGFP and *neo *could be used as two independent expression cassettes integrated into the same vector, this kind of approach could not guarantee EGFP and *neo *simultaneous expression. G418 drug selection could favour *neo *expression respect to EGFP, leading to a certain number of stably transfected, G418-resistant but not green EADSCs. Therefore, two different strategies were applied. First, an expression vector, pCMV-EGFP/*neo *(Fig. 5B), was generated by fusing the EGFP ORF in frame with the *neo *ORF downstream the hCMV promoter. A second expression vector, pCMV-EGFP-IRES-*neo *(Fig. 5C), containing an Internal Ribosomal Entry Site (IRES) that allows the simultaneous translation of two proteins (in the present case EGFP and *neo*) from a single transcript, was generated. Equal number of EADSCs were electroporated (see materials and methods) with equal amounts of pCMV-EGFP/*neo*, pCMV-EGFP-IRES-*neo *and pSP72 (a vector lacking both EGFP and *neo*) as a mock-transfected control. Following electroporation, cells were left to recover overnight and drug selection was applied with 400 μg of G418/ml of complete medium. Selected G418-resistant cells were counted when all mock-electroporated EADSCs died (Fig. 5A). As shown in Fig. 5B and 5C, pCMV-EGFP/*neo *leaded to a higher number of both G418 resistant and green EADSCs, compared to EADSCs electroporated with pCMV-EGFP-IRES-*neo *(Fig. 5B and 5C). Furthermore, pCMV-EGFP/*neo *stably transfected EADSCs maintained osteogenic properties as well as EGFP expression when induced to differentiate (the experiments were repeated for three times, giving similar results) (Fig. 5D). State of differentiation was confirmed with both alizarin and von Kossa staining (Fig. 6). Thus, electroporated GFP/neo fusion peptide along with hCMV promoter could be considered an efficient and versatile system to generate stably labelled EADSCs to be used *in vivo*.

### Paracrine delivery of secreted protein by transgenized EADSCs *in vivo*

Taking into account EADSCs capability to stably allocate a transgene *in vitro *without detrimental effect on their physiology, such characteristic was further investigated *in vivo*.

**Figure 7:**
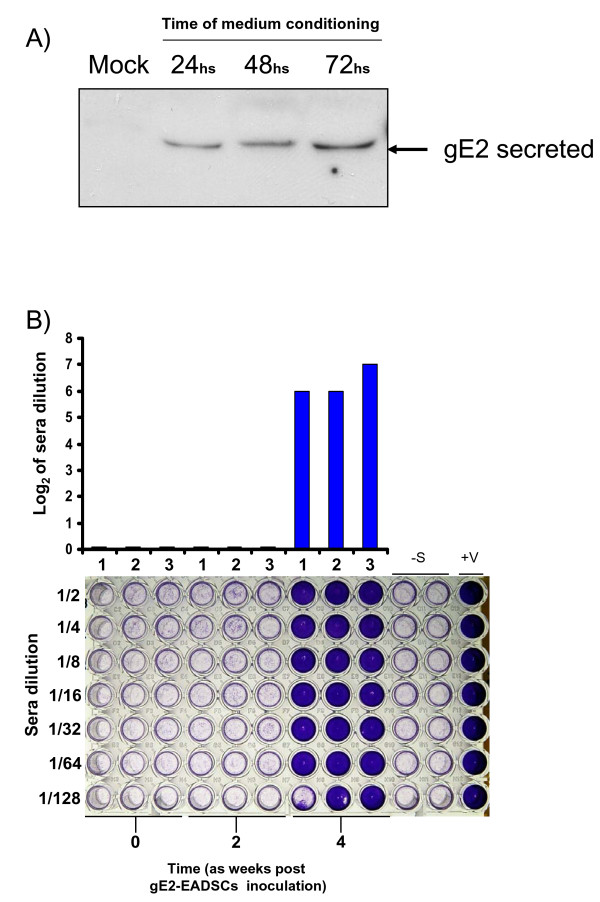
**Paracrine delivery of secreted protein by transgenized EADSCs *in vivo***. A) Western immunoblotting at 24, 48 and 72 h of gE2-EADSCs conditioned medium and compared to 72 h conditioned medium coming from mock-transfected and selected EADSCs. B) Humoral immune responses of 3 horses (1, 2 and 3) inoculated with gE2-EADSCs. Preimmune sera (time 0), sera at 2 weeks post gE2-EADSCs inoculation (time 2) and sera at 4 weeks post gE2-EADSCs inoculation (time 4 or time 2 from the second post gE2-EADSCs inoculation). Anti BVDV antibodies were detected by serum neutralization (SN) test. SN antibodies were expressed as the reciprocal of the highest dilution of the serum that inhibited the development of virus-induced CPE in MDBK cells. Virus neutralization (VN) titers of >1 (log_2_) were considered to be positive. Each value represents the response of each horse. Multiwells plate image with respective horse sera dilution, where crystal violet staining allows macroscopic evaluation of the integrity (violet wells) or the destruction (clear transparent wells) of the cell monolayer. The test was repeated three times and the same result was obtained. Controls were established in the absence of sera (-S) or with sera coming from BVDV infected animals.

As a general method to demonstrate the capability of transgenized EADSCs to deliver a potential secreted factor, an immunodominant secreted antigen previously shown to elicit a strong immune response was adopted [7]. EADSCs were transfected with a construct, pSecE2 [7], expressing the viral secreted antigen Bovine Viral Diarrhea Virus glycoprotein E2 (BVDV gE2) and EADSCs stably expressing the secreted antigen (gE2-EADSCs) were generated as previously described [8] (Fig. 7A). Immediately after the collection of the preimmune serum, 3 horses were intramuscularly inoculated with 3 × 10^6 ^gE2-EADSCs. An identical inoculation was performed 2 weeks later. Blood samples were collected at 2 weeks following each inoculum from all animals for the assessment of antibodies against BVDV gE2. Furthermore, body temperature and development of any adverse reaction to the inoculum were monitored daily. None of the animals developed fever or other clinical signs during the time of observation (4 weeks). All animals developed a serum neutralizing antibody response against BVDV, detectable the fourth week after the first viral inoculation (Fig. 7B). It was therefore possible to conclude that EADSCs were able to express and deliver an exogenous secreted factor, in the present case a viral antigen, able to elicit the humoral immune response into the inoculated animals.

## Discussion and Conclusions

Adipose-derived cells differentiate into several cell types [9]. Although it is not clear if a single adipose-derived cell can differentiate into all of these lineages, in different labs ADSCs clones expressing four cell lineages (adipo-, chondro-, osteo-, and neuro-) [10] have been generated, thereby demonstrating the presence of multipotent and oligopotent cells within adipose tissue. Self-renewing and multipotent adipose-derived progenitor and stem cells have been shown to be the ideal candidates for the generation of induced pluripotent stem (iPS) cells by transduction with four standard reprogramming factors, c-Myc, Klf4, Oct4, and Sox2 [11], [12]. Moreover, ADSCs exhibit high intrinsic expression of self-renewal supporting factors and can effectively serve as feeder layers of their own or as independent pluripotent cells [11], [12]. Since Equine iPS (EiPS) have not been generated so far, methods to genetically manipulate EADSCs would be of great interest for a future generation of EiPS. Therefore, the genetic manipulation of EADSCs represents a prerequisite for the above-listed purposes.

Transcriptional activity of different promoters was tested by dual luciferase reporter assay and hCMV promoter was proven to be the better transcribed by EADSCs. Although gene delivery was achieved with high efficiency by a VSV-G pseudo-typed self-inactivating replicating-incompetent third-generation lentiviral vector, as well as by BoHV-4-based vector, a safer physical method to stably transfect EADSCs was assessed, to avoid the concerns attributed to the use of integrating and non-integrating viral vectors. For instance, it is well known that integrating lentiviral vectors could lead to insertional mutagenesis, whereas the un-integrating BoHV-4 induced a strong cytopathic effect on EADSCs due to high viral replication. Therefore, electroporation with optimized parameters was employed and shown to be as good as viral transduction in term of gene delivery efficiency. Moreover, successfully transduced or transfected EADSCs maintained their differentiating capability when induced to differentiate toward the osteogenic lineage. Osteogenic differentiation property is widely accepted as the biological read-out for the differentiation of cells with a mesenchymal derivation, as is the case for EADSCs.

Expressing multiple transgenes within the same target EADSCs could be required for multiple gene transfer and EADSCs-based therapy application as well as for tracking EADSCs engraftment and differentiation within a specific area of the animal body. Gene function studies are best performed by expressing cDNAs together with a marker gene; by means of this approach, genetically modified EADSCs can be identified *in vitro *and *in vivo*. Similarly, transgenized EADSCs-based therapy applications can be improved by purification of gene-corrected EADSCs before *in vivo *administration, taking advantage of coordinate expression of drug selectable markers or living colour reporter genes. Coordinate expression of more than one transgene in EADSCs is essential when the activity to be reconstituted by gene transfer depends on multiple subunits encoded by different genes, or requires the synergism of separate molecules. The co-expression of *neo *and EGFP as a fusion protein (EGFP/*neo*) in EADSCs was successfully obtained, since EADSCs were simultaneously labelled and selected in a stable form and with high efficiency, compared to the inefficient IRES strategy, in which re-initiation of the translation of the second cistron, in this case the *neo *ORF, was highly inefficient and leaded to a low number of drug selected EADSCs. However, a further alternative approach could be employed, which is based on 2A self-cleaving peptides of the Foot and Mouth Disease Virus (FMDV) and other picoRNA-viruses [13]; however, application of this technology has been limited until recently because it requires molecular engineering of both transgenes and introduces sequence changes that may affect the activity, stability and immunogenicity of their protein product.

MSCs that act as Trojan horses to deliver therapeutic factors could be one step forward to gene delivery in suitable diseases, for instance joint- and teno-ligament disorders and EADSCs seem to have all the characteristics for such kind of objective. As a matter of fact, stably transfected EADSCs with secreted antigen were able to immunize horses when EADSCs were intramuscularly injected, showing that any kind of potentially therapeutic secreted factor could be delivered by EADSCs upon transgenesis. For instance, neurotrophic and trophic factors (such as brain-derived neurotrophic factor, glial cell line-derived neurotrophic factor (GDNF), ciliary neurotrophic factor, vascular endothelial-derived growth factor, insulin-like growth factor-1 (IGF-1) and Bone Morphogenic Proteins (BMPs) have been demonstrated promising in recovery or delaying the onset of many diseases [14]. In the same manner, the use of phage display-based single-chain miniantibodies (scFvs), which are small and consist of a single fusion polypeptide, has been applied. ScFvs are often derived from natural or synthetic cDNA libraries, but they can also be engineered from the sequences encoding the variable regions of individual hybridomas. In the latter case, scFvs retain the antigen binding properties of the parent monoclonal antibody [15]. However, clinical trials using neurotrophic factors or scFvs have been unsuccessful thus far because of problems with drug pharmacokinetics, dose-limiting toxicities and the potential for antibody inactivation. Therefore, significant efforts have focused on optimal delivery methods for these factors. Several studies have investigated pump-based delivery of recombinant proteins or direct gene delivery by viral and non-viral vectors, although with contrasting results, thus the transplantation of EADSCs expressing these factors could represent an alternative and possibly winning strategy. Interestingly, many works demonstrated that ADSCs not genetically-altered to express specific factors also showed a trend for increased neuromuscular function and increased survival, potentially through trophic factors that ADSCs themselves naturally provide [16], [17]. Further study of EADSCs based on these results is certainly warranted to advance their clinical application in individuals with specific diseases, as is the case of joint diseases and tendon injuries in horses. However, several key questions remain open: how many injections will be needed in a patient? Will the therapy be a one-time delivery? Will the cells survive, integrate, and express for long periods of time in animal tissues? Will the cells migrate to other organs? In any case, this work represents an additional step for the development of therapeutic approaches for cell-based therapy in the horse itself and in the horse as a model for human diseases.

## Methods

### ADSCs preparation

Cells preparation was performed as previously described with minor modifications [18]. Adipose tissue (approximately 15-20 grams) was collected from intra-abdominal fat deposit or supragluteal subcutaneous region of adult horses at the abattoir immediately after slaughtering. Tissue was minced into small pieces under sterile conditions and washed several times with phosphate buffer saline solution (PBS) containing penicillin (1,000 IU/ml) and streptomycin (100 mg/ml) (Sigma-Aldrich Corp., ST. Louis, MO, USA). The tissue was then digested with 7.5 mg/ml type I collagenase (Sigma-Aldrich) in Dulbecco's modified Eagle's medium low glucose (DMEM) (Lonza, Inc. Rockland, ME, USA) medium under mild shaking for 1 hour at 37°C. After digestion, the remaining tissue fragments were removed, cells suspension was filtered through a 50 μm nylon mesh and centrifuged at 250 × g for 10 minutes. The supernatant was removed and the cell pellet resuspended in DMEM containing 10% v/v fetal bovine serum (FBS) (Lonza), 1,000 IU/ml penicillin and 100 mg/ml streptomycin. Finally cells were diluted to 5.0 ×10^4 ^cells/cm^2 ^in culture medium, seeded in 25 cm^2 ^tissue culture flasks and incubated at 37°C in 5% CO_2_. After 48 h the medium was changed with fresh medium to remove non adherent cells; thereafter, medium was renewed every 3 days. The first subculture was performed after 7-10 days. Subsequent subcultures were performed when cells reached about 80% confluence.

### Constructs design and generation

pHSV-TK luciferase, pCMV-luciferase, pSV40-luciferase, pPGK-luciferase and pTK-renilla were obtained from Promega (Promega Corp., Madison, WI, USA). pBoHV-4 IE2 was constructed by sub-cloning a PCR fragment, corresponding to the BoHV-4 Immediate Early 2 (IE2) promoter in front of the luciferase reporter gene contained into the pGL3-basic vector as previously described [19]. To obtain pEGFP-IRES-neo, the EGFP ORF was cut with NheI and BamHI and legated into the NheI/BamHI sites of pIRES*neo*2 (Clontech Laboratories Inc., Mountain View, CA, USA). Whereas, to get pCMV-EGFP/*neo*, neo ORF was amplified with a couple of primers (neo/KpnI/sense, 5'-cgg ggt acc atg att gaa caa gat gga ttg-3' and neo/KpnI/antisense, 5'-cgg ggt acc tca gaa gaa ctc gtc aag aag-3') and the resulting amplicon sub-cloned in frame to the carboxy-terminal of EGFP into the pEGFP-C1 vector (Clontech Laboratories). Whereas, pSecE2 was obtained sub-cloning the Bovine Viral diarrhea Virus glycoprotein E2 into the pSecTag2 vector (Invitrogen Corp., Carlsbad, CA, USA) as previously described [7].

### Dual luciferase reporter assay

Confluent EADSCs in twenty-four well plates were co-transfected with 0.5 μg of reporter construct or 0.5 μg pGL3 empty vector, as a negative control and 0.05 μg of pTK-Renilla to normalize the efficiency of transfection, using LTX transfection reagent (Invitrogen) as suggested by the manufacturer. Transfection mixture was prepared in DMEM without serum and antibiotics and left on the cells for 6 h at 37°C, 5% CO_2 _in air, in a humidified incubator. After 6 h, the transfection mixture was replaced with complete medium (RPMI-1640 (Lonza), 10% FBS, 50 IU/ml of penicillin, 50 μg/ml of streptomycin and 2.5 μg/ml of Amphotericin B (Sigma-Aldrich)) and left to recover for 18 h at 37°C, 5% CO_2 _in air, in a humidified incubator. Twenty-four hours post transfection, cells were analyzed for Luciferase expression.

Luciferase reporter assay was performed with a Dual Luciferase Reporter Assay System kit (Promega) with minor modifications. Following treatments, cells were washed with PBS, lysed with 100 μl of lysis passive buffer by freeze-thawing at -80°C. 10 μl of the cell lysate were added to 50 μl of LAR and Luciferase activity was determined with a PerkinElmer Victor^3 ^Multilabel Counter (PerkinElmer, Waltham, MA, USA), according to the manufacturer's specifications. Individual assays were normalized for *Renilla *activity with a second reading, adding 50 μl of Stop & Glo substrate (Promega). Experiments were performed with 4 replicates at each time point and each experiment repeated three times. Statistical differences were tested by ANOVA.

### EADSCs transfection

*Lypofection: *was performed using LTX transfection reagent (Invitrogen) as suggested by manufacturer and described above, the only difference was that reagents were scaled-up to 25 cm^2 ^flasks. *Calcium phosphate precipitation*: was performed as previously described by others [20]. *Electroporation: *EADSCs were sub cultured to a fresh 25 cm^2 ^flask when growth reached 90% confluence and were incubated at 37°C in a humidified atmosphere of 95% air-5% CO_2_. Plasmid DNA (5 μg) in 600 μl DMEM without serum was electroporated (Equibio apparatus, Wolf Laboratories Limit., York, UK), 186 V, 960 μF, 4-mm gap cuvettes (Wolf Laboratories)) into EADSCs. Electroporated cells were returned to a new flask containing complete medium.

### Stable transfection

EADSCs were electroporated as described above. Electroporated cells were returned to the flask, fed the next day with complete medium supplemented with 400 μg/ml of G418 (Sigma-Aldrich) and split 1:2 when they reached confluence at 2 days post electroporation. Cells were grown till complete selection.

### BoHV-4-based vector preparation, infection and recovery assay

Infection of EADSCs was performed with 1 MOI (multiplicity of infection) of BoHV-4EGFPDTK, which was propagated as previously described [21]. A BT cell line (bovine turbinate cells, ATCC, CRL-1390, LGC Standards S.r.l., Milano, Italy) was used to propagate the virus, because of its high sensitivity towards infection of BoHV-4. BT cells were cultured in DMEM containing 10% FBS, 2 mM L-glutamine, 100 U/ml penicillin and 100 μg/ml streptomycin, and incubated at 37°C in a humidified atmosphere containing 5% CO_2_. BoHV-4EGFPDTK was propagated by infecting confluent monolayers with 1 of 50% tissue culture infective dose (TCID_50_) per 5 cells. After 4 days, cultures were frozen and thawed three times to release the virus. After removal of cell debris by low-speed centrifugation, the TCID_50 _was determined by limiting dilution. Virus stock thus obtained was stored at -80°C.

Cell cultures in six-well plates were infected with virus at a MOI of 10 TCID_50_/cell for 1 h. After infection, the inactivation of extracellular virus was carried out by low-pH treatment [22]. Briefly, the medium was removed and the plates were washed once with PBS, and then incubated for 2 min with either PBS (control plates) or a buffer (pH 3) containing 40 mM citric acid (Sigma-Aldrich), 10 mM KCl (Sigma-Aldrich), and 135 mM NaCl (Sigma-Aldrich). This procedure completely inactivated the absorbed non-penetrated infectious particles. Cultures were washed with medium and cultured till CPE completely destroyed the cell monolayer, after which 1 ml of the medium was removed from each well and centrifuged for 5 min at 1000 × g in a bench-top centrifuge to remove any cellular debris. TCID50 were determined on MDBK cells by limiting dilution.

### Lentiviral vector preparation and transduction of EADSC

Third generation of lentiviral vector were prepared as previously described [23]. Seventy-five cm^2 ^flasks of 293T cells were transfected by calcium-phosphate precipitation based method. Two hours before transfection, growing media (DMEM, 10% FBS, 50 IU/ml of penicillin, 50 μg/ml of streptomycin and 2.5 μg/ml of Amphotericin B) were changed with the fresh ones and four plasmids DNA transfection mix were prepared by adding 9 μg of envelope plasmid (pMD2-VSVG), 12.25 μg of core packaging plasmid (pMDLg/pRRE), 6.25 μg of REV plasmid (pRSV-REV) and 25 μg of transfer vector (pCCLsin18.PPT.Prom.GFP.Wpre). The plasmid solution was made up to a final volume of 500 μl with 450 μl of H_2_O and 50 μl of 2.5 M CaCl_2 _(Sigma-Aldrich). The precipitate was formed by drop wise addition of 500 μl of the 2× HBS solution (280 mM NaCl (Sigma-Aldrich), 100 mM HEPES (Sigma-Aldrich), 1.5 mM Na_2_HPO_4 _(Sigma-Aldrich), pH 7.12) to the 500 μl plasmid DNA-CaCl_2 _mix and finally added to the 293T cells. The Ca-Phosphate-DNA precipitate was allowed to stay on the cells for 14-16 h, after which the medium was replaced with 10 ml of fresh medium. Forty-eight hours after changing the media, the cell supernatant containing viral particles was collected, centrifuged at 500 × g for 5 minutes, filtered through a 0. 22 μm nitrocellulose filter, aliquoted and stored at -80°C. Transducing units on ml (TU/ml), were determined by limiting dilution on 293T cells.

EADSCs were plated on 25 cm^2 ^flasks and grown till they reached 80% (~ 10^6 ^cells) of confluence. The lentiviral vector was added onto each flask at an MOI of 1. EADSCs were incubated overnight at 37°C and then the media was replaced with 5 ml of fresh media. After 2 days of incubation, cells were analyzed for EGFP expression under a fluorescence microscope or flow cytometry.

### Flow cytometry

Transfected or transduced EADSCs, were then washed twice with PBS and 5 μl of 7-Amino-actinomycin D (7-AAD, BD Biosciences, San Jose, CA, USA) solution were added to asses cell death. Cells were acquired by FACSCalibur flow cytometer (BD Biosciences) equipped with a 15 mW Argon laser using 488 nm band pass filter, calibrated with CALIBRITE 3 beads (BD Biosciences) and FACSComp Software (BD Biosciences). Analysis were performed using Cell Quest software (BD Biosciences).

### ADSCs osteogenic differentiation and specific staining

For inducing osteogenic differentiation mesenchymal stem cells were plated at a density of 5,000 cells/cm^2 ^in a 25 cm^2 ^culture flask. When cells reached about 80% of confluence they were treated with dexamethasone (Sigma-Aldrich) (0.1 μM), β-glicerophospate disodium (Sigma-Aldrich) (10 mM) and ascorbic acid (Sigma-Aldrich) (250 μM) in DMEM with 10% FBS. After 10-15 days, osteogenic differentiation was demonstrated by von Kossa silver reduction method and alizarin staining. The differentiated cultures were fixed 20 minutes at room temperature with a PBS 4% paraformaldehyde (Sigma-Aldrich) solution. Cells were washed twice with distilled water and then stained either with alizarin or von Kossa method. For the von Kossa staining, the von Kossa Bioptica kit (04-170801) (Bioptica s.p.a., Milano, Italy) was used, as described by the supplier. Briefly, cells were incubated subsequently in lithium carbonate solution, silver nitrate solution, reducing solution and sodium sulphite solution (provided by the kit) to determine the specific substitution of calcium ions by cationic silver. Nuclei were counterstained with Mayer's Carmalum (Bioptica).

For alizarin red staining, the StemPro Osteogenesis Differentiation kit (Invitrogen) was employed as described by the supplier. Fixed cells were washed twice with distilled water and incubated with 2% Alizarin Red S solution (pH 4.2), provided by the kit, for 2 to 3 minutes. Subsequently, stained cells were washed three times with distilled water and visualized under a light microscope and images were captured for qualitative analysis.

### Western immunoblotting

Protein free cell supernatants (20 μl) were electrophoresed through 10% SDS-polyacrylamide gels and transferred to nylon membranes by electro-blotting. Membranes were incubated with monoclonal anti-BVDV-gE2 (clone 157; VRMD, Inc., Pullman, USA), probed with horseradish peroxidase-labeled anti-mouse immunoglobulin antibody (Sigma-Aldrich), and visualized by enhanced chemiluminescence (ECL Kit; PIERCE, Rockford, IL, USA).

### Serological tests

Serum neutralization tests were performed as follows. Twenty-five microliters of each serum sample were added to the first line of wells of 96-well plates. Twenty-five microliters of DMEM were added to each well and, for each serum tested, serial two-fold dilutions were made. Positive and negative serum controls were included. Twenty-five microliters of virus suspension containing 100 TCID50 of BVDV was added to each well. After 1 h of incubation at 37°C, 50 μl of a MDBK cell suspension were added to each well and the plates were incubated for 3 days at 37°C in a humidified atmosphere of 95% air-5% CO_2_. Expression of viral infectivity and serum neutralizing activity through CPE were detected by microscopy and or by crystal violet (Sigma-Aldrich) staining of the cell monolayer. The neutralization antibody titers were expressed as the reciprocal (log 2) of the final dilution of serum that completely inhibited viral infectivity.

### Animal handling and care

Horses were cared for and used in accordance with Italian laws for animal experimentation. Horses were maintained at 24°C with a controlled light cycle (12 h of light, starting at 6:00 a.m.) and with food and water ad libitum. Blood samples were obtained and viral injections were performed via the auricular vein at scheduled intervals.

## Authors' contributions

GD: Conceive the experiments, performed the experiments and wrote the paper. GM: performed animal experiments. AC: contributed to perform the experiments. VF: contributed to perform the experiments. VC: performed cell culture and staining. SG: contributed to conceive and perform the experiments and wrote the paper. All authors read and approved the final manuscript.
